# A call for re-evaluation of the guidelines for prophylactic ICD implantation

**DOI:** 10.1007/s12471-014-0591-3

**Published:** 2014-08-29

**Authors:** J. G. L. M. Luermans, M. Mafi Rad, K. Vernooy

**Affiliations:** Department of Cardiology, Maastricht University Medical Center, Maastricht, PO BOX 5800, 6202 AZ the Netherlands

The implantable cardioverter-defibrillator (ICD) is the most effective therapy currently available to prevent sudden cardiac death (SCD) in patients with left ventricular dysfunction.[1–4] The current ACC/AHA/ESC guidelines for prophylactic ICD implantation in patients with left ventricular dysfunction [5] are based on large randomised clinical trials performed in the 1990s and the beginning of this century.[1–4] However, these guidelines might be out of date since the treatment of patients with coronary artery disease (CAD) and dilated cardiomyopathy (DCM) has improved considerably in the last decade. Moreover, long-term complications of ICD implantation have become more apparent. Real-world follow-up data of ICD implantations in patients who received an ICD based on the current guidelines are needed to determine whether the guidelines are still applicable and risk factors should be identified differentiating patients who do or do not benefit from prophylactic ICD therapy.

In this issue of the Netherlands Heart Journal Verhagen and co-workers report their retrospective follow-up data of ICD implantations in patients with impaired left ventricular function due to CAD or DCM. [6] The study population consisted of 320 CAD and 178 DCM patients who received an ICD with or without cardiac resynchronisation therapy (CRT) between January 2005 and June 2012 according to the current guidelines. Overall mortality of the total study population was 14.5 % (72 patients), during a median follow-up of 40 months with no significant differences between CAD and DCM patients. At 1, 2 and 5 years, mortality rates were 2.2 %, 4.2 % and 13.5 %, respectively. These relatively low mortality rates are in agreement with Smith et al. who reported a mortality of 7 %, with no differences between CAD and DCM patients, during a median follow-up of 31 months, in a recently published Dutch registry in 290 CAD and 137 DCM patients with ICD implantations between 2004 and 2009. [7] At 1, 2 and 5 years, mortality rates were 2.4 %, 4.3 % and 14.7 %. The mortality rates shown by Verhagen et al. and Smith et al. are considerably lower when compared with the landmark MADIT-II (16 % at 2 years) (2) and SCD-HeFT (29 % at 5 years) [3] trials and are probably the result of the improvements that have been made over the years in the treatment of (ventricular arrhythmias in) ischaemic heart disease and dilated cardiomyopathy.

According to the current ACC/AHA/ESC guidelines for management of patients with ventricular arrhythmias and the prevention of sudden cardiac death, left ventricular dysfunction (i.e. left ventricular ejection fraction (LVEF) ≤ 30-35 %) and functional impairment as classified by the New York Heart Association (NYHA) are the two main parameters determining the indication for prophylactic ICD implantation in these patients.[5] Despite the fact that LVEF and NYHA functional class are powerful predictors of the risk of death, these parameters have limitations in predicting death due to cardiac arrhythmias. As the NYHA functional class and LVEF worsen, total mortality increases, although the proportion of total deaths due to cardiac arrhythmias decreases.[8] A better risk stratification is needed in order to select patients who benefit most from ICD therapy. Determining the LVEF in combination with other arrhythmia risk factors might improve the accuracy of predicting sudden death due to cardiac arrhythmias. Verhagen et al. report by multivariate analysis that impaired LVEF, age ≥ 75 years, QRS > 120 ms, and renal insufficiency were independent predictors for mortality.[6] This is in agreement with Goldenberg et al. who proposed a five risk factor clinical model in their post hoc analysis of the MADIT II trial.[9] Based on these five risk factors (age > 70 years, renal insufficiency, atrial fibrillation, NYHA class > 2, and QRS duration > 120 ms) they were able to identify patients with reduced benefit of ICD implantation because of either too low or too high risk. However, stronger evidence is needed before such risk factors are implemented in the guidelines.

The cumulative incidence of appropriate shocks in the study by Verhagen et al. was 4.4 %, 7.2 % and 13.1 % at 1, 2 and 5 years follow-up with no difference between the two groups.[6] This incidence is lower than for instance in the SCD-HeFT tria l [3] and can largely be explained by the improvements in device programming in the last decade.-[10, 11] Also, inclusion of CRT ICDs and developments that have been made over the years in the treatment of the underlying heart disease and the treatment of ventricular arrhythmias might account for the lower incidence of appropriate shocks. However, we would have expected other predictors for appropriate shocks than the use of digoxin and a history of smoking found by Verhagen et al [6].

The cumulative incidence of inappropriate shock therapy was 5.4 %, 7.2 % and 9.0 % at 1, 2 and 5 years follow-up with no significant differences between CAD and DCM patients. [6] Most inappropriate shocks were caused by atrial fibrillation, both paroxysmal and permanent atrial fibrillation. An incidence of inappropriate shocks of up to 21 % has been reported in primary prevention trials.[4, 10] The reduction in the incidence of inappropriate shocks over the years is probably grossly the result of improvements in device technology, algorithms and programming.[11, 12].

Obviously, and as the author’s already describe, the study by Verhagen et al. has some important limitations. The study group is heterogeneous, with large differences between the CAD and DCM groups and differences in follow-up duration between patients. This is inherent to the retrospective character of the study. Moreover, one-third of the included patients received CRT and it remains unclear whether they responded to CRT or not. This might have influenced their results remarkably. Additionally, the cause of death of the deceased patients and the device-related complications have not been reported. These data would have been interesting, since then the yield of ICD implantation could be better weighed against the risks.

In conclusion, the results of the study performed by Verhagen et al. demonstrate that the current mortality rate of patients undergoing prophylactic ICD implantation is much lower as compared with the older landmark trials. Therefore, the current guidelines for prophylactic ICD implantation might need reconsideration. We need to re-evaluate the efficacy of ICD implantation and to better identify patients who do not benefit from ICD implantation, since it involves increased risks for these patients and high costs. Verhagen et al. provide us with a prelude to the DO-IT trial. The DO-IT trial is a Dutch national, multicentre prospective ICD registry study, which started enrolment in January 2014. Approximately 1500 patients will be included in the registry in the participating high-volume ICD centres in the Netherlands. The objective is to evaluate the practice of ICD implantation in the Netherlands and to identify patients who will not benefit from ICD implantation for primary prevention of SCD within two-years follow-up.
